# Maturation of Pain Empathy from Child to Adult Shifts from Single to Multiple Neural Rhythms to Support Interoceptive Representations

**DOI:** 10.1038/s41598-018-19810-3

**Published:** 2018-01-29

**Authors:** Jonathan Levy, Abraham Goldstein, Maayan Pratt, Ruth Feldman

**Affiliations:** 1 Interdisciplinary Center, Herzliya, 46150 Israel; 2 Gonda Multidisciplinary Brain Research Center, Bar-Ilan University, Ramat Gan, 5290002 Israel; 3 Department of Psychology, Bar-Ilan University, Ramat Gan, 5290002 Israel; 4 Yale University, Child Study Center, New Haven, CT 06520 USA

## Abstract

While empathy to the pain of conspecific is evolutionary-ancient and is observed in rodents and in primates, it also integrates higher-order affective representations. Yet, it is unclear whether human empathy for pain is inborn or matures during development and what neural processes underpin its maturation. Using magnetoencephalography, we monitored the brain response of children, adolescents, and adults (n = 209) to others’ pain, testing the shift from childhood to adult functioning. Results indicate that children’s vicarious empathy for pain operates via rudimentary sensory predictions involving alpha oscillations in somatosensory cortex, while adults’ response recruits advanced mechanisms of updating sensory predictions and activating affective empathy in viceromotor cortex via higher-level representations involving beta- and gamma-band activity. Our findings suggest that full-blown empathy to others’ pain emerges only in adulthood and involves a shift from sensory self-based to interoceptive other-focused mechanisms that support human altruism, maintain self-other differentiation, modulate feedback to monitor other’s state, and activate a plan of action to alleviate other’s suffering.

## Introduction

Empathy is a multifaceted phenomenon most commonly considered from the perspectives of shared affect or cognitive mentalization^1–3^; the first being more rudimentary and evolutionary-ancient, the second more advanced and human-specific^3,4^. Research on the shared affective representations of empathy has typically been conducted by investigating empathy to vicarious physical pain^1–3^. Empathy for pain, a capacity sculpted by the long history of mammalian evolution, enhance species survival by increasing brain sensitivity to the pain of kin and affiliates and expanding threat-detection to the level of the group thereby motivating care for conspecifics^5,6^. Yet, while rudimentary empathy for pain is associated with sensory processing and observed in rodents^7^ and primates^8^, it also extends to include higher-order affective representations in addition to sensorimotor activations^9^, efficiently differentiates self from other^10^, expands from kin to humankind, and integrates sensory-motor resonance with cognitive understanding of others’ needs and emotions^11^. However, despite extant research on empathy for pain and its neural correlates^1–3,10,12^, it is unclear whether the mature form of human empathy for pain is inborn or develops over time and what neural processes underpin its maturation.

Developmental studies indicate that rudimentary empathy is observed already in newborns, expressed as resonance to another infant’s cry^13^, and develops across childhood and adolescence, reaching maturity in adulthood^14^. Neuroimaging studies report that this early empathy relies on sensory and rudimentary networks, which are mostly in place by the end of infancy, whereas mature empathy recruits frontal areas that reach maturity by late adolescence or young adulthood^15,16^. Singer and colleagues^17^ showed that mature human empathy for pain taps into frontal viceromotor regions, including the anterior insula (AI), anterior cingulate cortex (ACC) and orbitofrontal cortex (OFC), reflecting affect understanding and higher-order representations. Such viceromotor networks enable adults to draw upon the experience of one’s own bodily milieu, i.e., interoception, in representing another person’s physical pain or mental state^18^.

Oscillations are a highly-conserved and pervasive feature of neuronal activity^19^ and probing changes in neural oscillations provides efficient route to understanding brain functioning beyond anatomy, particularly network interactions underlying cognitive functions^20^. The maturation of neural oscillations may mirror developmental stages. For instance, research in humans and animals suggests that gamma-band activity does not emerge before maturity^21–23^, and since gamma integrates higher-order information in viceromotor regions during pain perception^24–27^, it may present a powerful developmental marker that signals the shift to higher-level interoceptive processing. According to the predictive coding frame, neural rhythms capture distinct functions; alpha underpins the construction of predictions, beta involves updating predictions, while gamma implicates prediction errors^28^. Furthermore, the gamma-beta interplay enables an efficient forward-backwards flow^29^, and the suppression of alpha activity activates such efficient gamma-beta exchange^28,30,31^. Thus, at a rudimentary level, alpha oscillations may sustain somatosensory representations through feedback information, while at higher level, gamma oscillations may optimize learning via feedforward routes that rely on the transfer from superficial cell populations which send prediction errors via gamma activity before passing to deeper layers to code predictions via alpha and beta mechanisms^32,33^.

Extant evidence suggests that alpha-^34–36^ and beta-band^34,37^ activity in sensorimotor and primary somatosensory cortex (S1) underpins empathy for pain. Yet, very little is known about the developmental course of these rhythms during empathy for pain processing. Generally, alpha is considered the predominant oscillation in the awake human brain and is viewed as the default rhythm^38^, while beta implicates higher-order processes such as prediction^39^, confidence^40^ and gain control^28^. With regards to empathy, alpha may support self-based sensations, whereas beta may underpin error-monitoring, which is critical for other-focused empathy^41^. Taking into consideration the link between the expression of gamma oscillations and maturity, it is plausible to expect a developmental progress from alpha to beta to gamma from children to adolescents to adults.

**Figure 1:**
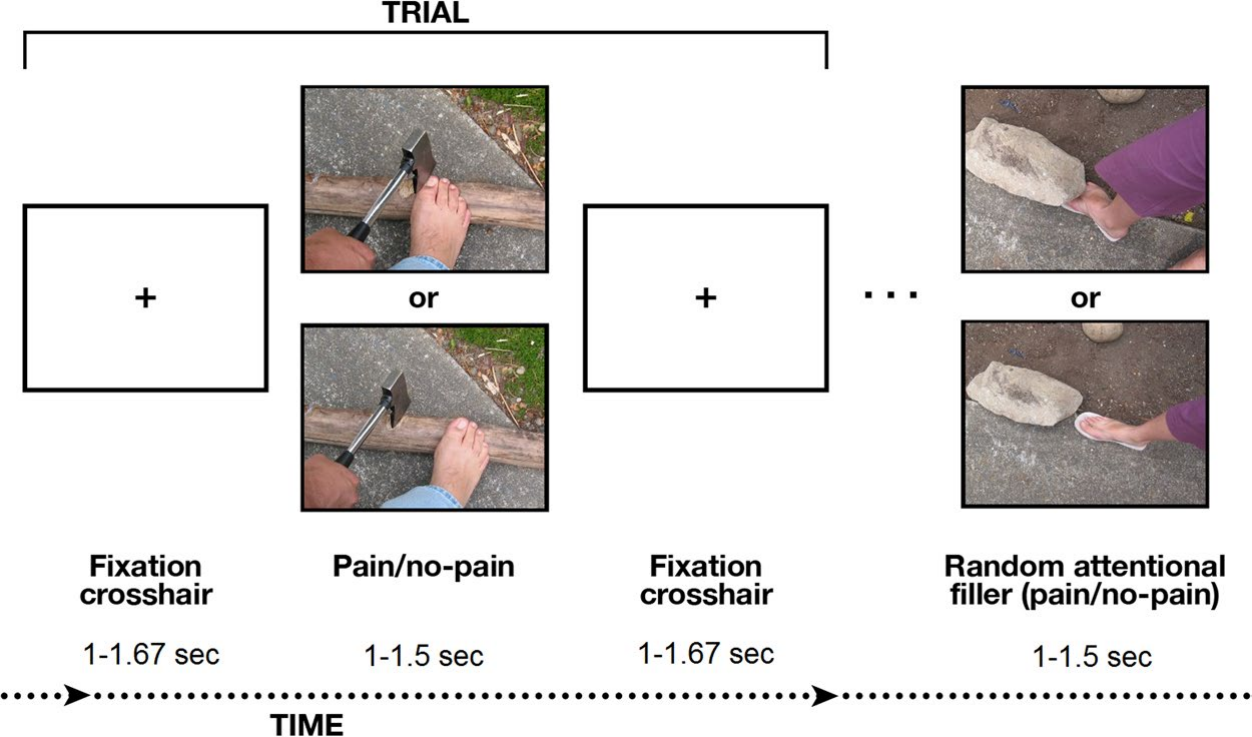
Experimental procedure describing the Pain (P) and no-Pain (no-P) stimuli presented, with the random attentional fillers illustrating a twirl in the pictures.

The current study employed magnetoencephalography (MEG) to monitor oscillatory brain responses in children, adolescents, and adults to others’ pain. As empathy for pain is one of the most basic, rudimentary and evolutionary-ancient components of empathy, we employed the well-validated semi-passive paradigm involving the visual perception of pictures depicting human targets under physical pain^42^, as illustrated in Fig. 1. The advantage of an approach involving passive perception is that it taps directly into the automatic neural processing of empathy for pain without implicating non-specific processes such as cognitive, decision-making, or motor responses to the stimuli^34,43,44^.

We hypothesized that the maturation and integration of multiple neural rhythms could sustain the building of full-blown empathy for pain to other’s suffering and that this shift may be coupled with the transition from sensory self-based to evaluative other-focused functioning. As alpha is the predominant oscillation in the awake brain, we expected alpha to sustain empathy for pain across development. However, as humans transit into adulthood, the brain may incorporate beta and gamma rhythms, integrating lower- and higher-order empathic representations and shifting from sensory-based to viceromotor representational processing.

## Results

### MEG-array sensor-level results

**Figure 2:**
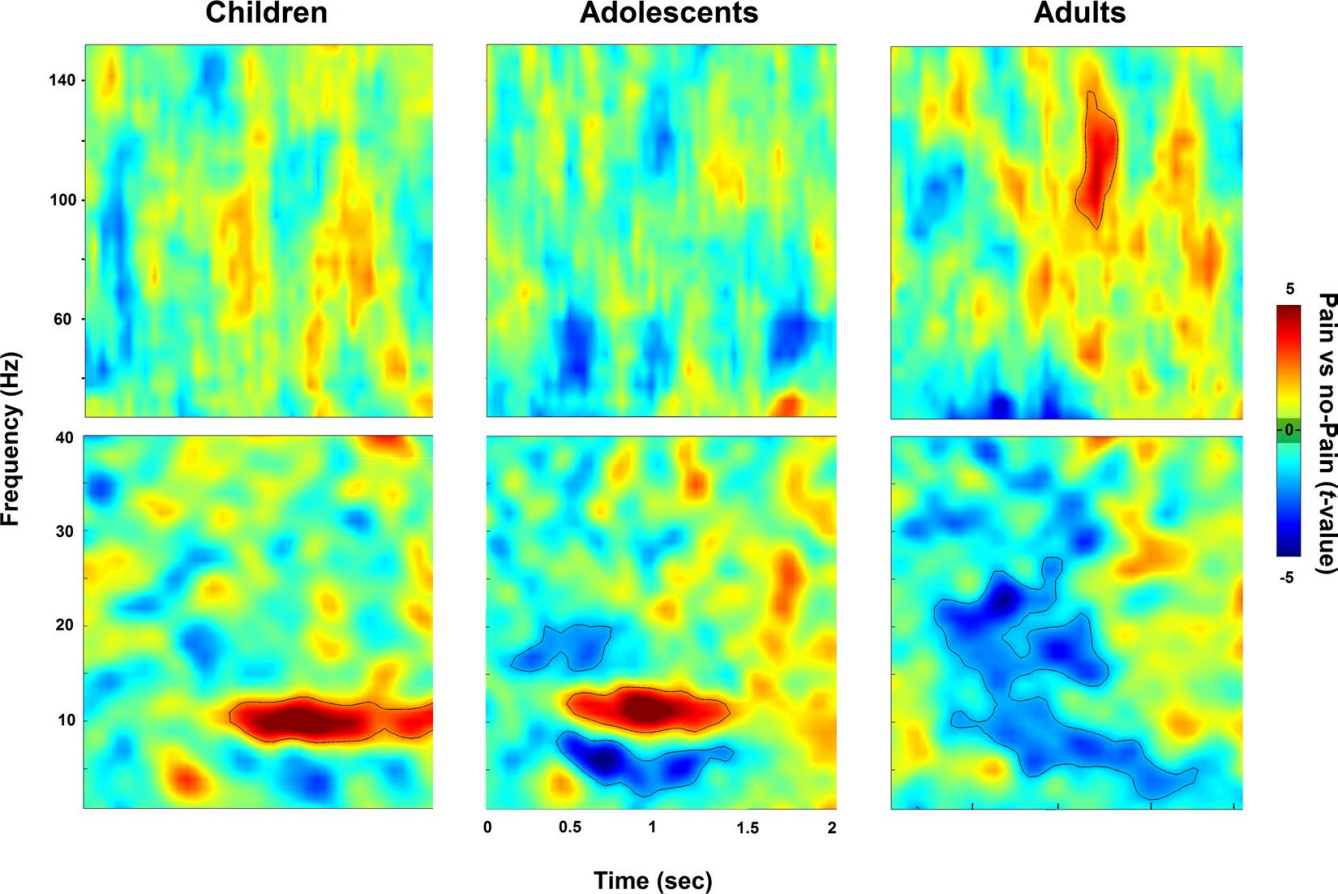
Sensor-level spectral maps conveying empathy for pain. The statistical maps of Pain vs no-Pain stimuli averaged above all sensors in children (n = 85), adolescents (n = 80) and in adults (n = 44). Lower panels describe < 40 Hz frequencies which were calculated with a hanning taper, in comparison to the Slepian multitapers used for the upper panels. Contoured patterns illustrate statistically significant time-frequency windows (p_cluster-cor_ < 0.05).

Participants watched a set of well-validated visual stimuli depicting limbs in painful or non-painful conditions^42^ while we measured ongoing neural oscillatory activity using MEG (see Fig. 1). The detection rate in the attentional filler task (see Fig. 1, right panel) was high (ca. 90%) without statistically significant difference (p = 0.11) between children and adults. The post-scan rating of the level of pain depicted in the stimuli was overall very high (M = 4.30, SD = 0.89) without any statistically significant (p = 0.70) difference between the children and the adults groups. We then probed the neural effect of empathy for pain (Pain vs no-Pain) at the whole MEG sensor-array level. The statistical time-frequency maps (0–2.3 sec; 1–150 Hz) in the three developmental groups are represented on Fig. 2, with statistically significant time-frequency patterns (*P*_cluster-cor_ < 0.05) contoured within a black line. Children exhibited a statistically significant time-frequency pattern expressed as late sustained high alpha (peaking at ~10 Hz) (see Fig. 2, left panels). By contrast, adolescents kept a pattern of alpha band enhancement, similar to that of children; yet at the same time, they exhibited a statistically significant pattern of delta, theta, low alpha (peaking at ~7 Hz) and beta suppression patterns (see Fig. 2, middle panels). Finally, adults exhibited a statistically significant pattern of beta, low alpha (peaking at ~8 Hz), theta and delta suppression, similar to adolescents, but they lacked the alpha enhancement pattern present in the children and adolescents groups. Furthermore, adults presented a distinctive oscillatory pattern in the high frequencies: a broad band gamma power enhancement (see Fig. 2, right panels).

We then proceeded to test whether these apparently distinct developmental patterns are reflected at the between-group statistical level. We focused on alpha, beta and gamma band activity, as much less is known about theta and delta in the context of empathy for pain. At the alpha-band, there was no statistically significant difference (*P*_cluster-cor_ = 0.22) in high-alpha enhancement between children and adolescents. Yet, there was a statistically significant difference (*P*_cluster-cor_ < 0.001) between adolescents and adults, such that adults had significantly stronger alpha suppression. Likewise, there was a statistically significant difference (*P*_cluster-cor_ < 0.001) between children and adults, such that adults had significantly stronger alpha suppression. At the beta-band, there was no statistically significant difference (*P*_cluster-cor_ = 0.72) between children and adolescents. Yet, there was a statistically significant difference (*P*_cluster-cor_ < 0.05) between adolescents and adults, such that adults had significantly stronger beta suppression. Likewise, there was a statistically significant difference (*P*_cluster-cor_ < 0.01) between children and adults, such that adults had significantly stronger beta suppression. At the gamma-band, there was no statistically significant difference (*P*_cluster-cor_ = 0.57) between children and adolescents. Yet, there was a statistically significant difference (*P*_cluster-cor_ < 0.01) between adolescents and adults, such that adults had significantly stronger gamma enhancement. Likewise, there was a statistically significant difference (*P*_cluster-cor_ < 0.05) between children and adults, such that adults had significantly stronger gamma enhancement.

The time-frequency patterns that were obtained by comparing among groups were remarkably similar to those obtained by comparing P vs no-P stimuli in each group separately. For instance, the gamma-band effect was obtained when contrasting adults and adolescents or adults and children; this effect was also obtained when contrasting P vs no-P effect for each group. Noteworthy, the experimental design of the adolescents group included an additional component of group-membership, and this may have biased the results. Yet, we previously found that the group-membership prime changed the intensity of the alpha effect but did not affect the observed oscillatory patterns^45^; still, it is suggested that the adolescent findings should be interpreted with caution and guide future research. Hence, taken together, these sensor findings suggest a possible gradual oscillatory pattern which is triggered by empathy for pain as a function of age group: alpha (enhancement) in children, alpha (enhancement and suppression) and beta suppression in adolescents, and suppression of alpha and beta coupled with enhancement of gamma-band activity in adults.

### Controls

Despite these remarkable developmental findings, it should be noted that there were two parameters which were not equally matched in the three age groups: gender and stimulus duration. Children and adolescents were well-matched (56.25% and 47.5% female proportions, respectively), but adults consisted only of females. Although the comprehensive meta-analysis on empathy for pain^12^ did not find gender differences in any empathy for pain study, this is a study limitation. As for stimulus presentation time, 40 of the children and all adults had stimuli presented for 1 sec, whereas 45 of the children all adolescents had stimuli presented for 1.5 sec. To attempt to control for these biases, we compared between the gender samples in the children and the adolescents groups. The alpha effect in children was not statistically significant affected by gender (*P*_cluster-cor_ = 0.20). The alpha effect in children was not significantly affected by gender (*P*_cluster-cor_ = 0.20). In the adolescents group, the alpha effect was not significantly affected by gender (*P*_cluster-cor_ = 0.58), nor was the beta effect (*P*_cluster-cor_ = 0.26).

Furthermore, to have a supplementary control of whether male participants may have biased the findings, we reanalysed the data while excluding all male participants. The alpha-band suppression effect between adolescents and adults was maintained (*P*_cluster-cor_ = 0.007) as well as between children and adults (*P*_cluster-cor_ = 0.004). The beta-band suppression effect between adolescents and adults was not maintained (*P*_cluster-cor_ = 0.21) *when* applying the conservative approach of localizing a time-frequency window; but directly testing the specific beta effect (obtained in the gender mixed sample) yielded a significant effect (*P* = 0.04) also in the female sample. The beta-band suppression effect between children and adults (*P*_cluster-cor_ = 0.03) was also maintained.

The gamma-band enhancement effects between adolescents and adults (*P*_cluster-cor_ = 0.05) as well as between children and adults (*P*_cluster-cor_ = 0.03) were both maintained.

To address the difference of stimulus duration, we compared the samples of children with stimulus duration of 1.5 s to those with 1 s. There was a statistically significant difference (*P*_cluster-cor_ < 0.005), but this reflected differences later than 1.8 s, which was marginal in the bulk of the alpha enhancement window, and was outside of the between-groups comparison effect.

As for a second control of the possible influence of stimulus duration, we repeated the analyses while examining only the first 1 s post stimulus onset as for to rule out possible effects due to the presentations exceeding 1 s. Noteworthy only the beta-band effect was entirely restricted in the first 1 s, whereas the effect in the alpha-band was extending from before to after 1 s, and the gamma-band effect emerged completely after 1 s and therefore could not be reanalysed. The reanalysis constrained within the first 1 s yielded the following: The alpha-band suppression effect (the part of it constrained within the first 1 s) between adolescents and adults was maintained (*P*_cluster-cor_ = 0.001) as well as between children and adults (*P*_cluster-cor_ = 0.002). The beta-band suppression effect between adolescents and adults was maintained (*P*_cluster-cor_ = 0.01) as well as between children and adults (*P*_cluster-cor_ = 0.001). Certainly, a better balance between the age groups on gender and stimulus duration would be best. Yet our supplementary analyses suggest that these parameters probably do not affect the extent of the developmental findings reported here.

### Cortical source-level results

**Figure 3:**
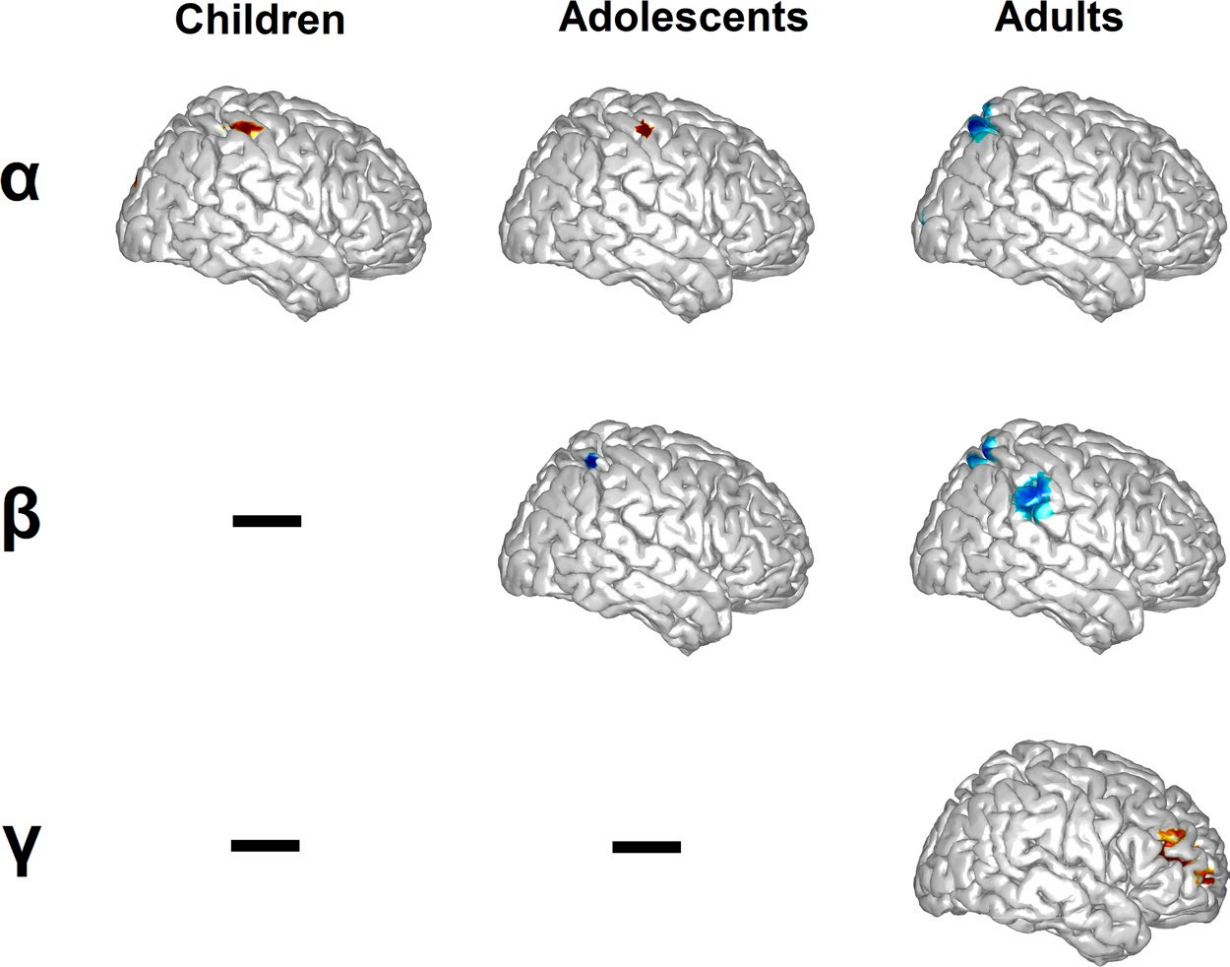
Age-dependent source-level localization of spectral patterns in alpha, beta and gamma. The Pain vs. no-Pain contrast is laid over age-averaged MNI templates. Colors on the templates represent peak statistical activity (p_cluster-cor_ < 0.05).

Next, we probed whether such developmental recruitment of increasingly more rhythms may match a functional cortical recruitment, as previously found^16,46^. Source localization (masked at *P*_cluster-cor_ < 0.05) revealed that the (alpha-band) oscillatory pattern in children emanated particularly from bilateral S1 and the central sulcus (see Fig. 3, left upper panel); this pattern was strikingly similar in the adolescents group (see Fig. 3, middle upper panel). Similarly, in the adults group, the source peaked in the bilateral S1, with a cluster extending into the parietal cortex (see Fig. 3, right upper panel). Further, source localization for the beta-band in the adolescents group revealed peak activity in the bilateral parietal lobe with a cluster extending into S1, and including also the middle cingulate cortex (see Fig. 3, middle panel). This pattern was very similar in adults, where it peaked in the parietal lobe with a cluster extending into the bilateral S1 (see Fig. 3, right middle panel). Finally, source localization for the gamma-band (in the adults group) revealed left-hemispheric peak in the viceromotor (VM) cortex (see Fig. 3, right lower panel). The source findings in adults support previous evidence of alpha and beta oscillations sustaining sensorimotor function during empathy for pain^34–37^ and extends it by reporting that gamma-band activity represents the shift to the higher-order functioning of other-focused empathy for pain in the VM^17^. Moreover, the findings reveal that development of empathy for pain entails the transition from the sensory to higher order function, and that this leans on an oscillatory shift from a single-rhythm template to a multi-rhythm and efficient interplay.

## Discussion

The current study focused on a fundamental social process, empathy for pain, a highly-conserved social phenomenon that supports group cohesion and enhances survival in mammals but also underpins humans’ unique ability to represent others’ state and share their suffering. Notwithstanding its evolutionary roots, we followed the maturation of empathy for pain in children, adolescents, and adults, consistent with Tinbergen’s proposal that ontogenetic assessment is a requirement when describing a phenomenon from an evolutionary perspective^47^. Utilizing magnetoencephalography on a large sample covering a wide age-range, we tested for the first time the maturation of neural oscillations and their cortical generators sustaining human empathy for pain and addressed their ontogenetic roots. We found that while both children and adults rated the stimuli as very painful and their behavioural rating was comparable, neural response to vicarious pain showed a marked developmental progression with age. Specifically, we found that in processing vicarious empathy for pain the brain makes two important shifts from its childhood to adult format: shifting from a single-rhythm template to a multi-rhythm efficient interplay, and expanding from a sensorimotor neural response to additionally activating the affective empathy network. These neural modulations at the levels of both substrate and rhythmic activity may be taken as indicators of a developmental transition from a rudimentary self-based to complex other-centred processing of empathy for pain as individuals mature from childhood to adolescence to adulthood. Our findings that empathy for pain shifts from an exteroceptive process based on sensory input from the environment to an interoceptive mechanism supported by representations of one’s own bodily experiences can therefore shed new light on the neural basis of human empathy for pain.

Our findings suggest that empathy for pain develops along a gradual course from sensory alpha band enhancement, through sensory alpha band suppression coupled with beta band modulation, and culminating in viceromotor gamma band activity. Thus, a uni-rhythm alpha response in childhood gradually integrates beta in adolescence and gamma in adulthood into a multi-rhythm, excitatory-inhibitory exchange operating across brain sites implicated in sensorimotor processing, affect salience, embodiment, and interoceptive representations. This progression involves the gradual orchestration of several rhythms, distinct neural networks, and mechanisms of enhancement with those of suppression. Of note, maturation of the full-blown human empathic response is a very long process, not completing its course by 18 years of age. It has been suggested that humans’ protracted maturity enables the great plasticity of the human brain and its impressive capacity to adapt to multiple ecologies and integrate contextual determinants into its own functioning^48,49^. Thus, while empathy for pain is highly-conserved, its slow development affords incorporation of contextual, affective, representational, and future-directed elements into its unique expression by human adults. This process may also render the mature human empathy for pain a vulnerable achievement, open to conditions such as early adversity^50^ or outgroup derogation^45^.

Tracking empathy for pain across development, we found that to generate empathy, children rely on sensory alpha oscillations. However, with the transition into adulthood, this pattern of sensory alpha oscillations is gradually supressed. Past electrophysiological research has mainly tested empathy for pain in adults and the few studies using MEG reported alpha suppression in S1^34,36^, consistent with our findings. Very little research tested oscillatory patterns of empathy for pain in children; yet, Cheng and colleagues using EEG and could not detect alpha suppression above central sensors^51^. Possibly by using MEG and extracting S1 source data, future studies may be able to detect alpha oscillations that sustain empathy for pain in children. At present, the functional interpretation of the developmental transition from alpha enhancement to alpha suppression is not fully clear and requires much further research. Yet, our findings clearly demonstrate that regardless of age group, the brain responds very strongly to vicarious pain and development induces qualitative changes in this response, for instance, the transition from alpha enhancement to suppression. Much further research is required to uncover the mechanisms underpinning the maturation of neural responses to fundamental social stimuli, such as empathy for pain, and studies utilizing the same paradigm across a wide age range are very much in need in social neuroscience.

In addition to the suppression of alpha, the transition into the mature adult format of empathy for pain was marked by beta modulations, and this appears to be present already in adolescence. Very few studies investigated the role of beta in empathy for pain and, in general, less is known about the functional role of beta oscillations. Yet, our results are consistent with two prior studies which showed that empathy for pain in adults elicits beta suppression above central sensors, probably reflecting sensorimotor activity^34,37^. Other lines of research point to the role of beta in higher-order functions involving precision and gain control. Within the predictive coding frame beta is considered a mechanism for detecting post synaptic gains in neuronal sensitivity in neurons reporting on predictions and determining information flow towards higher-order targets for further processing^28,40,52^. While much further research is needed to understand the function of beta oscillations, the present results add a developmental angle by demonstrating that beta gradually emerges during adolescence on top of the childhood alpha as a mid-step towards mature empathic processing.

Gamma band activity serves as a developmental marker of brain maturation in humans and other mammals^21–23,53,54^. Furthermore, gamma in prefrontal and viceromotor regions is thought to integrate higher-order information^55–59^. This suggests that the viceromotor gamma activity found here in a network supporting interoceptive representations may reflect the understanding that the other, not the self is in pain and the recruitment of embodiment and affective salience mechanisms to gauge and partake in others’ distress. We suggest that in the case of empathy for pain, gamma oscillations fine-tune the affective sharing and cognitive processing of another human’s pain on the basis of one’s own bodily self^60^. Recent advances in the study of interoception suggest that the agranular viceromotor cortex generates predictions about the expected state of the body which are constrained by sensory input^18^. Our findings corroborate previous lines of research and suggest that viceromotor gamma may signal a transition from sensory self-based to representational other-focused empathy and that such transition mirrors human developmental stages.

Research on pain perception within the predictive coding frame indicates that pain perception is critically determined by predictions and their updating through learning with an interplay between low- and high-frequency oscillations^32,33^. Adapted into the present context, empathy for pain may rely on sensory predictions implicating alpha in the immature brain while development gradually updates these sensory predictions via beta modulations that gauge precision of incoming stimuli by means of post-synaptic “broadcasting”^61^, and finally culminating in the recruitment of selective anterior empathy nodes for grounding experience in the present moment, interoceptive sensitivity^61^, and fine-tuning the affective magnitude of the experience via prediction error gamma. Such interpretation of our findings requires further validation and should be treated with caution.

Very little research followed the neural basis of empathy from childhood to adulthood and none utilized magnetoencephalography, and thus, our findings integrating oscillatory rhythms and their cortical sources across development can shed new light on the evolution of the human-specific form of empathy from its evolutionary-ancient origins. We show that unlike developmental milestones which are highly canalized (e.g., motor development^62^), empathy for pain reaches its maturity through a process that gradually unfolds with children’s emerging representational capacities and integrates contextual information that enhances, for good or for bad, human fittedness to their social ecology. We found that mature empathy for pain recruits the self’s deep representations of bodily states (e.g., thermoregulation, proprioception, nociception, energy balance) in the service of sharing other’s pain. Thus, the shift from self-based sensory processing to interoceptive representations is not trivial and marks a human achievement sculpted by a long evolutionary history that is susceptible to a range of biological and environmental risks. Much further research is required to assess maturation of the neural basis of empathy for pain across cultures, contexts, and psychopathological conditions and understand the ways by which our brain enables a meaningful participation in the distress of others.

## Materials and Methods

### Participants

Two hundred and nine participants were recruited to study the neural response to empathy for pain, including children (n = 85, 45 females; M ± SD, 11.42 ± 1.05), adolescents ((n = 80, 38 females; M ± SD, 16.63 ± 0.89),) and adults (n = 44, all females; M ± SD, 41.35 ± 4.53). The study received approval from the Bar-Ilan University ethics committee, and informed consent was obtained from all participants (and from the parents of the minor participants) who have received monetary compensation for their participation in the study. All experiments were performed in accordance with the relevant guidelines and regulations.

### Experimental Design

#### Stimuli

We programmed and operated the experiment using E-Prime® software (Psychology Software Tools Incorporated). We used two types of stimuli: pain (P) and no-pain (no-P) stimuli^42^. All stimuli appeared in uniform size (300 × 225 pixels) at the center of a gray background on a 20-inch monitor, at a viewing distance of approximately 55 cm. A series of 96 digital color pictures showed limbs (right hands and right feet) in P (48 stimuli) and no-P (48 stimuli), at a ratio of 51/49% for legs/hands. The purpose of the P stimuli was to elicit empathy for pain, while that of no-P stimuli was to control for the other parameters induced by the visual stimuli.

#### Procedure

Participants lay in supine position inside the MEG system while facing a screen projecting the stimuli. Subjects received instructions to remain relaxed, not move their limbs and to watch the presented stimuli. The experimenters observed their compliance using an infrared camera. The stimuli presented while measuring participants’ brain activity comprised 110 trials [or 180 trials in a sub-sample (n = 45) of the children sample; 288 trials in the adolescents data] per experiment. P and no-P stimuli were presented for 1 sec (or for 1.5 sec in the children n = 45 sub-sample and in the adolescents data) each, interleaved with crosshair fixation screens randomly varying in duration between 1 and 1.67 sec (for 1.5 sec in the children n = 45 sub-sample). It is noteworthy that the adolescents sample was reanalyzed from a previous study^45^ to probe empathy for pain towards ingroup targets. Similar to previous studies^34,45,50^, in order to maintain and assess attentional resources throughout the session, we randomly inserted attentional filler trials (ca. 11% of all trials) by creating a short twisted movement in new selected stimuli by using a twirl filter (Photoshop, Adobe Systems Inc.) – see static illustration of the twirl on the right panel of Fig. 1. Participants were instructed to press a button when detecting these stimuli and were trained on the task before the experimental session started. We did not include the filler trials in the experimental stimuli database or analyse them. After the scanning session, participants were debriefed about how they felt during the experiment, and asked to rate how painful (1–5) they found the painful images in the task.

### MEG recordings and data preprocessing

We recorded ongoing brain activity (sampling rate, 1017 Hz, online 1–400 Hz band-pass filter) using a whole-head 248-channel magnetometer array (4-D Neuroimaging, Magnes® 3600 WH) inside a magnetically shielded room. Reference coils located approximately 30 cm above the head, oriented by the x, y and z axes enabled removal of environmental noise. We analyzed data of 2300 ms epochs including a baseline period of 450 ms filtered in the 1–200 Hz range with 10 sec padding and then resampled them to 400 Hz.

### Spectral and source analyses

We attached five coils to the participant’s scalp to record head position relative to the sensor. We performed analyses using MATLAB 7 (MathWorks®, Natick, MA, USA) and the FieldTrip software toolbox^63^. We applied tapers to each time window to compute Time-Frequency Representations (TFRs) of power for each trial and to calculate the Fast Fourier Transform (FFT) for short sliding time windows. We analyzed data in alignment to stimulus onset and then averaged the power estimates across tapers. A Hanning taper, applied to each epoch of the 248-sensor data, yielded the FFT for short sliding time windows of 0.5 sec in the 1–30 Hz frequency range, resulting in a spectral resolution of 2 Hz. To probe gamma-frequency power (40–150 Hz), five Slepian multitapers^64^ were applied using a fixed window length of 0.2 s, resulting in a frequency smoothing of 15 Hz. We obtained induced activity by subtracting evoked-components’ power from oscillatory power. Finally, we determined time-frequency windows in which the P vs no-P contrast was statistically significant after correcting for multiple comparisons (see next section for more detail).

For source localization, head shape underwent manual digitization (Polhemus FASTRAK® digitizer), and a single shell brain model was built based on an MNI post-puberty template brain^65^, which we modified to fit each subject’s digitized head shape using SPM8 (Wellcome Department of Imaging Neuroscience, University College London, www.fil.ion.ucl.ac.uk). We then divided the subject’s brain volume into a regular grid, obtaining the grid positions by their linear transformation in a canonical 1 cm grid. This procedure facilitates group analysis, because it requires no spatial interpolation of the volumes on reconstructed activity. Finally, we used the statistically significant time-frequency windows obtained at the sensor level analyses to proceed with beamforming: For each grid position, we reconstructed spatial filters^66^ in the aim of optimally passing activity (in that time-frequency window) from the location of interest, while suppressing activity that was not of interest.

### Statistical Analysis

Statistical procedures on the MEG data assessed significance of the power values using a non-parametric approach^67^ which does take the cross-subject variance into account, because this variance is the basis for the width of the randomization distribution. This approach is valuable because it does not make any assumptions about underlying distribution and is unaffected by partial dependence between neighboring time-frequency pixels. Specifically, in the first step of the procedure we computed t-values per subject, channel, frequency, and time, representing the contrast between the conditions. Subsequently, we defined the test statistic by pooling the t-values over all participants. Here, we searched time-frequency clusters with effects that were significant at the random effects level after correcting for multiple comparisons along the time and the frequency dimensions. To compute the effect compared to baseline, the first step was replaced by adjusting the effect to the baseline level, and the second step applied a dependent t-test. These procedures would correspond to fixed-effect statistics, however, to make statistical inferences corresponding to a random effect statistic, we tested the significance of this group-level statistic by means of a randomization procedure: We randomly multiplied each individual t-value by 1 or by −1 and summed it over participants. Multiplying the individual t-value with 1 or −1 corresponds to permuting the original conditions in that subject.

We reiterated this random procedure 1000 times to obtain the randomization distribution for the group-level statistic. For each randomization, we retained only the maximal and the minimal cluster-level test statistic across all clusters, placing them into two histograms that we addressed as maximum/minimum cluster-level test statistic histograms. We then determined, for each cluster from the observed data, the fraction of the maximum/minimum cluster-level test statistic histogram that was greater/smaller than the cluster-level test statistic from the observed cluster. We retained the smaller of the two fractions and divided it by 1000, giving the multiple comparisons corrected significance thresholds for a two-sided test. The proportion of values in the randomization distribution exceeding the test statistic defines the Monte Carlo significance probability, which is also called a *P* value^67^. This cluster-based procedure allowed us to obtain a correction for multiple comparisons in all brain analyses.
